# Supplementary material for: Remote digital cognitive assessment for aging and dementia using the Oxford Cognitive Testing Portal OCTAL
Source: NPJ Digit Med. 2026 Jan 15;9:162. doi: 10.1038/s41746-026-02346-6 (PMC12909784; doi:10.1038/s41746-026-02346-6)
Supplement: Supplementary file 1 — Supplementary Information [file 41746_2026_2346_MOESM1_ESM.pdf]

Supplementary Materials

Supplementary Figure 1. Effect of first language status on OCTAL performance (Study 2, N = 1,109). __ 2

Supplementary Table 1: Demographic and cognitive performance comparisons between English- and Chinese-speaking participants (Study 1). ____ 4

Supplementary Figure 2: Rank-ordered Spearman correlations between screen width and OCTAL task metrics. ____ 6

Supplementary Table 2: Spearman correlations between screen width and OCTAL metrics. ____ 7

Supplementary Figure 3: Rank-ordered Spearman correlations between screen height and OCTAL task metrics. ____ 9

Supplementary Table 3: Spearman correlations between screen height and OCTAL metrics. ____ 11

Supplementary Figure 4: Two-factor structure derived from an exploratory factor analysis of 19 OCTAL metrics obtained remotely from 1,109 healthy participants across the adult lifespan (Study 2). ____ 13

Supplementary Table 4: Spearman correlations between age and OCTAL metrics in Study 2. ____ 14

Supplementary Figure 5: Age trajectories of OCTAL metrics in a healthy population (Study 2, N = 1,109). ____ 16

Supplementary Table 5: Slopes of age-effect on OCTAL metrics. ____ 17

Supplementary Figure 6. Scatter plots depicting the linear relation between age and each OCTAL metric. ____ 18

Supplementary Figure 7: Metric-by-metric correlations among OCTAL indices in healthy adults (Study 2), without multiple comparison correction. ____ 19

Supplementary Table 6 : Correlation table between ACE-III subscores. ____ 21

Supplementary Figure 8: ROC for different thresholding for ACE-III ____ 22

Supplementary Table 7: Consistency across different thresholding for ACE-III and task combinations for OCTAL. ____ 23

Supplementary Table 8: Intraclass correlation coefficients (ICCs) for all OCTAL and ACE-III metrics. 24

Supplementary Table 9: The full word corpus used in Verbal Memory Wordlist Recall Task (ALF). ____ 25

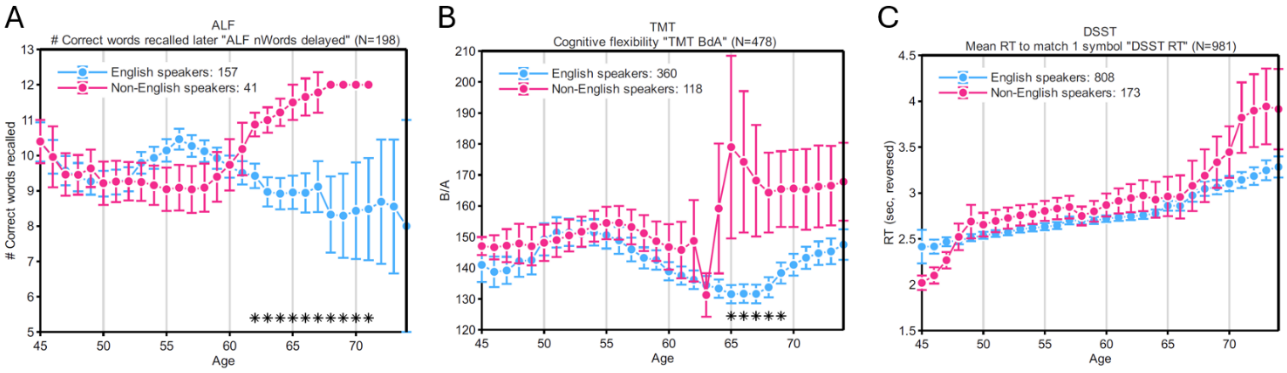

Supplementary Figure 1. Effect of first language status on OCTAL performance (Study 2, N = 1,109).

Scores for native English speakers are plotted in blue, and those for participants who use English as a second language are plotted in bright pink. Asterisks mark age intervals with significant group differences ($p < 0.05$). Error bars denote ± 1 SEM.

Does speaking English as a second language affect OCTAL performance?

We examined whether speaking English as a second language affects performance on OCTAL, a cognitive battery administered in English. In our previous study with a smaller sample of participants (Toniolo et al., 2024), we restricted participation to native English speakers. However, in practice—particularly in the UK—many patients maybe fluent in English but speak another language as their mother tongue. Although these individuals may function fluently in English, second-language use can require additional cognitive effort, particularly in verbal tasks. This raises the question: does not having English as a first language impact performance on OCTAL?

In this study, among the 1109 participants, 918 reported English as their first language, while 191 reported a different first language. We hypothesised that the verbal memory task (ALF)—which involves remembering a list of 12 common English words—would be the most susceptible to language-related differences. Surprisingly, no disadvantage was observed for non-native English speakers in either the immediate or delayed recall phases of the ALF task (**Figure 7A**). In fact, older adults whose first language was not English tended to perform slightly better in both recognition and recall. This may reflect our careful word selection process, which ensured that all ALF items were simple, high-frequency words.

Similarly, we found no evidence of a language effect on performance in other non-verbal tasks, including DSST, ROCF, OMT, and OIS, provided that participants could understand the instructions.

More nuanced effects emerged in the Trail Making Test (TMT). Language background had no impact on the control condition (connecting two dots) or TMT-A (connecting Arabic numerals in sequence), likely because Arabic numerals are universally taught in early education across languages and cultures. However, performance diverged in TMT-B, which requires alternating between numbers and letters (e.g., 1–A–2–B…). From around age 55 onwards, non-native English speakers performed significantly worse than their native English-speaking peers, even when taking TMT-A under control (**see Supplementary Figure 1 below**). Consequently, their TMT-B/A ratio—often used as an index of cognitive flexibility—appeared unreasonably low. This suggests a language-related bias in TMT-B, likely reflecting differences in alphabet familiarity or automaticity rather than true executive dysfunction.

These findings highlight the importance of considering language background when interpreting performance in language-loaded cognitive tasks, particularly when assessing executive function using TMT-B.

Supplementary Table 1: Demographic and cognitive performance comparisons between English- and Chinese-speaking participants (Study 1).

Demographic and cognitive performance of English- and Chinese-speaking participants (Study 1). This table extends Table 1 by reporting Mann–Whitney U statistics, effect sizes (r), and Bonferroni-corrected p values. Practical significance was evaluated with a bootstrap permutation procedure (10,000 iterations). For each metric, lower and upper thresholds were defined as 1 SD below and 1 SD above the mean of the English-speaking cohort, respectively. To determine whether the Chinese group (N = 210) differed meaningfully from these thresholds, N observations were resampled with replacement from the Chinese data, the mean was calculated, and this process was repeated 10,000 times. A difference was deemed significant when ≥97.5 % of resampled means lay below the lower threshold or above the upper threshold (p < 0.025).

	Mean (SD)		Mann-Whitney U			Bootstrap diff. from norm range			
Metric	English	Chinese	U	p-value	Effect Size r	Lower Threshold	Upper Threshold	Below -1SD	Above 1SD
N	194	167							
Gender (% Female)	44.9	57.5							
Age (years)	31.60 (6.88)	29.88 (5.72)	37252	p = 1.00	0.11	24.72	38.49	p = 1.00	p = 1.00
Education (years)	14.96 (2.02)	16.46 (1.52)	9613	p < 0.001	0.30	12.94	16.98	p = 1.00	p = 1.00
ROCF_CopyScore	99.07 (4.75)	99.26 (2.36)	34540.5	p = 1.00	0.01	94.33	103.82	p = 1.00	p = 1.00
ROCF_CopyDuration	105.87 (38.54)	117.85 (52.62)	32520.5	p = 1.00	0.11	67.33	144.4	p = 1.00	p = 1.00
ROCF_CopyMoveN	18.21 (5.46)	20.46 (8.73)	32460	p = 1.00	0.11	12.75	23.67	p = 1.00	p = 1.00
ROCF_RecallScore	92.36 (10.60)	95.07 (7.73)	32144	p = 0.69	0.13	81.76	102.95	p = 1.00	p = 1.00
ROCF_Remember	93.15 (9.38)	95.77 (7.36)	31600	p = 0.13	0.16	83.77	102.53	p = 1.00	p = 1.00
ROCF_RecallDuration	88.92 (39.91)	100.97 (56.53)	32282	p = 1.00	0.12	49.01	128.83	p = 1.00	p = 1.00
ROCF_RecallMoveN	17.65 (5.51)	19.28 (8.66)	32471	p = 1.00	0.11	12.13	23.16	p = 1.00	p = 1.00
OMT_Acc_Easy	96.95 (5.85)	98.86 (2.46)	20879	p < 0.01	0.23	91.1	102.8	p = 1.00	p = 1.00
OMT_Acc_Hard	85.88 (10.16)	89.96 (8.76)	20620	p = 0.01	0.21	75.72	96.05	p = 1.00	p = 1.00
OMT_LocErr_Easy	0.08 (0.05)	0.07 (0.03)	22946	p = 1.00	0.04	0.03	0.13	p = 1.00	p = 1.00
OMT_LocErr_Hard	0.16 (0.07)	0.15 (0.07)	24603	p = 1.00	0.07	0.09	0.23	p = 1.00	p = 1.00
OMT_Acc	91.42 (6.41)	94.41 (4.84)	19971	p < 0.001	0.25	85.01	97.82	p = 1.00	p = 1.00
OMT_IdeRT	1.73 (0.49)	2.28 (0.79)	17856	p < 0.001	0.41	1.24	2.22	p = 1.00	p = 0.16
OMT_LocErr	0.12 (0.05)	0.11 (0.04)	24167	p = 1.00	0.04	0.07	0.17	p = 1.00	p = 1.00
OMT_LocRT	2.10 (0.77)	2.50 (1.86)	22449	p = 1.00	0.10	1.32	2.87	p = 1.00	p = 0.99
OMT_TargetDetection	72.74 (13.84)	82.12 (12.55)	18953	p < 0.001	0.34	58.9	86.57	p = 1.00	p = 1.00
OMT_Misbinding	24.90 (10.91)	17.92 (11.88)	27612.5	p < 0.001	0.28	13.99	35.81	p = 1.00	p = 1.00
OMT_Guessing	14.73 (8.98)	8.88 (7.16)	29008	p < 0.001	0.36	5.75	23.71	p = 1.00	p = 1.00
OIS_STM_SemanticAcc	99.65 (2.86)	99.88 (0.77)	32330.5	p = 1.00	0.01	96.78	102.51	p = 1.00	p = 1.00
OIS_STM_Acc	97.33 (4.76)	97.49 (7.68)	31614.5	p = 1.00	0.05	92.57	102.08	p = 1.00	p = 1.00

OIS_STM_IdeRT	1.73 (0.24)	2.57 (0.92)	21094	p < 0.001	0.63	1.49	1.96	p = 1.00	***p < 0.001***
OIS_STM_LocErr	0.21 (0.05)	0.18 (0.05)	37636	p < 0.001	0.30	0.16	0.25	p = 1.00	p = 1.00
OIS_STM_LocRT	5.71 (0.39)	6.15 (3.64)	36199	p < 0.01	0.21	5.32	6.09	p = 1.00	p = 0.42
OIS_LTM_SemanticAcc	86.11 (16.75)	87.10 (15.28)	31867.5	p = 1.00	0.03	69.36	102.87	p = 1.00	p = 1.00
OIS_LTM_Acc	77.94 (19.79)	81.29 (18.42)	30666	p = 1.00	0.10	58.15	97.74	p = 1.00	p = 1.00
OIS_LTM_IdeRT	6.65 (1.90)	6.27 (1.82)	34419	p = 1.00	0.11	4.74	8.55	p = 1.00	p = 1.00
OIS_LTM_LocErr	0.21 (0.05)	0.20 (0.06)	32876	p = 1.00	0.03	0.16	0.26	p = 1.00	p = 1.00
OIS_LTM_LocRT	11.18 (1.12)	10.33 (3.49)	38231	p < 0.001	0.33	10.07	12.3	p = 0.84	p = 1.00
DSST_nCorrectResponse	52.11 (14.08)	51.88 (11.02)	24605.5	p = 1.00	0.05	38.03	66.19	p = 1.00	p = 1.00
DSST_RT	2.37 (2.27)	2.20 (0.47)	22408	p = 1.00	0.08	0.09	4.64	p = 1.00	p = 1.00
DSST_Accuracy	97.91 (3.57)	98.95 (1.90)	21283	p = 0.07	0.18	94.34	101.47	p = 1.00	p = 1.00
TMT_Connect2	3.72 (9.08)	2.56 (0.56)	31930.5	p = 1.00	0.10	-5.37	12.8	p = 1.00	p = 1.00
TMT_A	20.28 (9.24)	20.78 (7.10)	29531	p = 1.00	0.10	11.04	29.52	p = 1.00	p = 1.00
TMT_B	29.01 (11.54)	31.53 (9.09)	27852	p = 0.01	0.20	17.47	40.55	p = 1.00	p = 1.00
TMT_BdA	147.65 (35.30)	156.64 (40.80)	28977	p = 0.71	0.13	112.35	182.94	p = 1.00	p = 1.00

Supplementary Figure 2: Rank-ordered Spearman correlations between screen width and OCTAL task metrics.

*P values were Bonferroni-corrected for 35 comparisons (34 OCTAL metrics + age); ** denotes p < 0.01. The white numerals at the ends of bars gives the spearman rho values. All correlations were computed from a sample of N=210 in Study 1. The width of screen was captured and recorded automatically by the Credamo platform. The details of p values are available in **Supplementary Table 2**.*

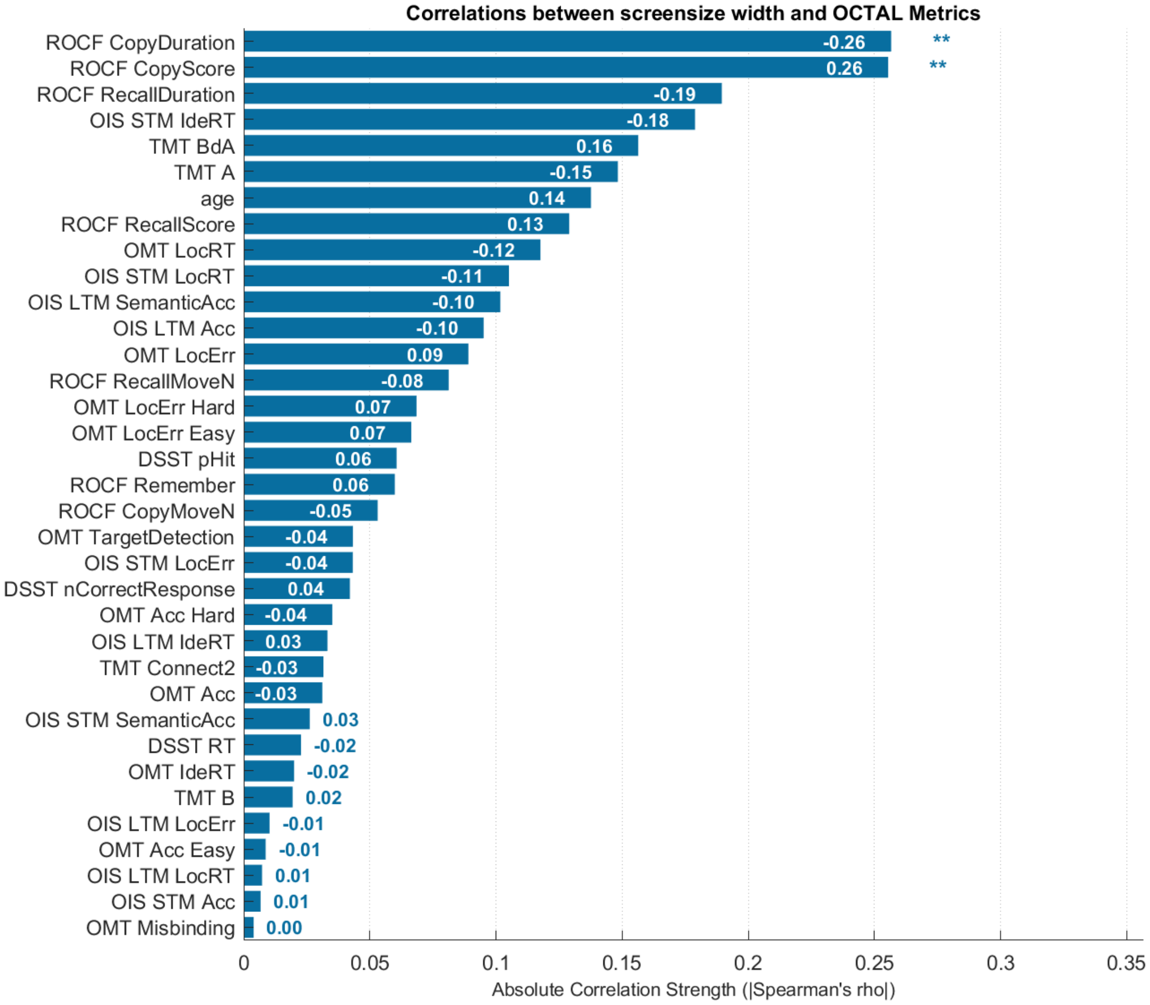

Supplementary Table 2: Spearman correlations between screen width and OCTAL metrics.

Metric	rho	pValue	Significance
ROCF_CopyDuration	-0.26	0.01	**
ROCF_CopyScore	0.26	0.01	**
ROCF_RecallDuration	-0.19	0.21	
OIS_STM_IdeRT	-0.18	0.33	
TMT_BdA	0.16	0.83	
TMT_A	-0.15	1.00	
age	0.14	1.00	
ROCF_RecallScore	0.13	1.00	
OMT_LocRT	-0.12	1.00	
OIS_STM_LocRT	-0.11	1.00	
OIS_LTM_SemanticAcc	-0.10	1.00	
OIS_LTM_Acc	-0.10	1.00	
OMT_LocErr	0.09	1.00	
ROCF_RecallMoveN	-0.08	1.00	
OMT_LocErr_Hard	0.07	1.00	
OMT_LocErr_Easy	0.07	1.00	
DSST_pHit	0.06	1.00	
ROCF_Remember	0.06	1.00	
ROCF_CopyMoveN	-0.05	1.00	
OMT_TargetDetection	-0.04	1.00	
OIS_STM_LocErr	-0.04	1.00	
DSST_nCorrectResponse	0.04	1.00	
OMT_Acc_Hard	-0.04	1.00	
OIS_LTM_IdeRT	0.03	1.00	
TMT_Connect2	-0.03	1.00	
OMT_Acc	-0.03	1.00	
OIS_STM_SemanticAcc	0.03	1.00	
DSST_RT	-0.02	1.00	
OMT_IdeRT	-0.02	1.00	
TMT_B	0.02	1.00	
OIS_LTM_LocErr	-0.01	1.00	

OMT_Acc_Easy	*-0.01*	*1.00*	
OIS_LTM_LocRT	*0.01*	*1.00*	
OIS_STM_Acc	*0.01*	*1.00*	
OMT_Misbinding	*0.00*	*1.00*	

Supplementary Figure 3: Rank-ordered Spearman correlations between screen height and OCTAL task metrics.

P values were Bonferroni-corrected for 35 comparisons (34 OCTAL metrics + age); denotes p < 0.05, *** denotes p < 0.001. The white numerals at the ends of bars gives the spearman rho values. All correlations were computed from a sample of N=210 in Study 1. The width of screen was captured and recorded automatically by the Credamo platform. The details of p values are available in **Supplementary Table 3**.*

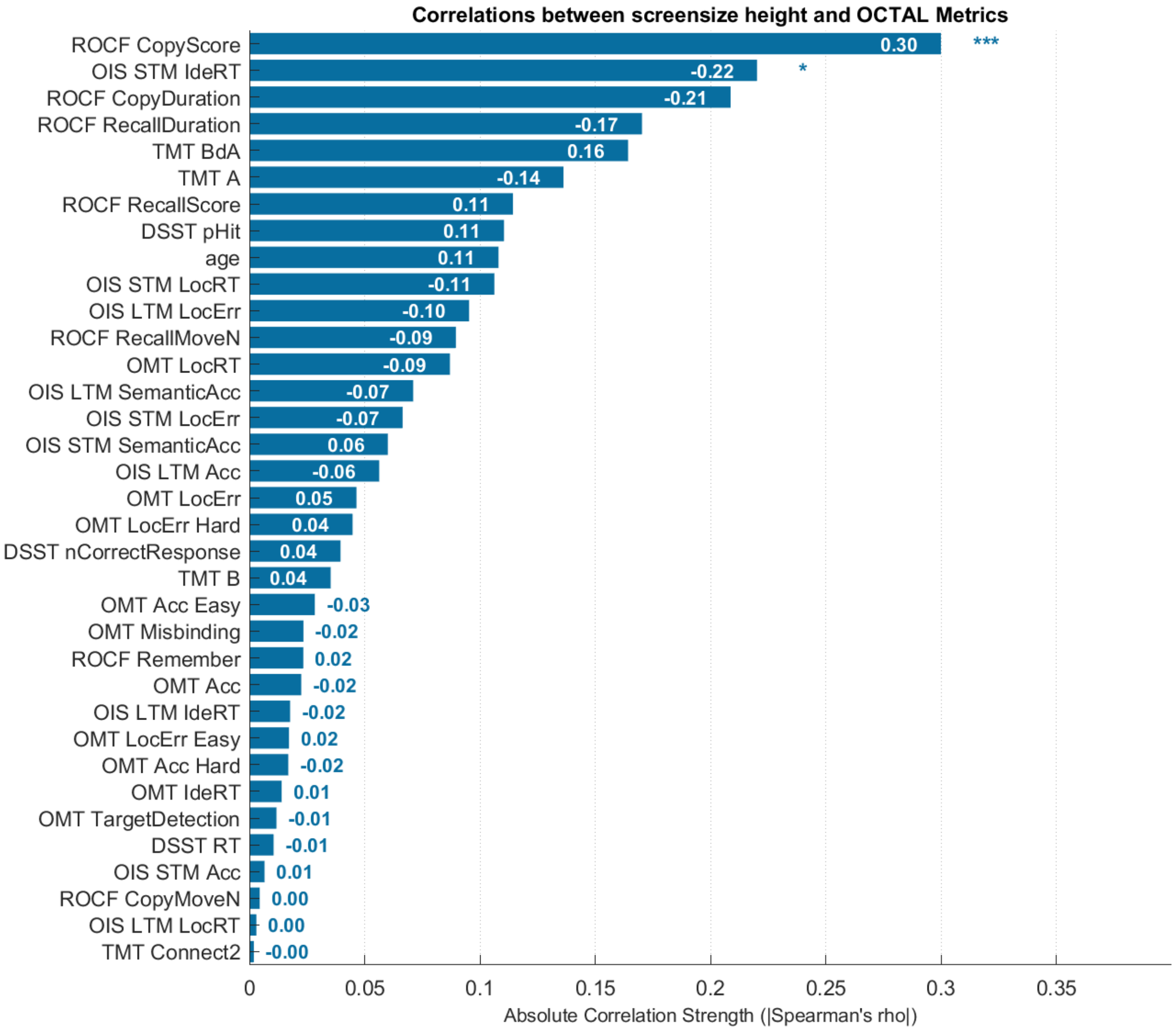
Correlations between screensize height and OCTAL Metrics
ROCF CopyScore 0.30 ***
OIS STM IdeRT -0.22 *
ROCF CopyDuration -0.21
ROCF RecallDuration -0.17
TMT BdA 0.16
TMT A -0.14
ROCF RecallScore 0.11
DSST pHit 0.11
age 0.11
OIS STM LocRT -0.11
OIS LTM LocErr -0.10
ROCF RecallMoveN -0.09
OMT LocRT -0.09
OIS LTM SemanticAcc -0.07
OIS STM LocErr -0.07
OIS STM SemanticAcc 0.06
OIS LTM Acc -0.06
OMT LocErr 0.05
OMT LocErr Hard 0.04
DSST nCorrectResponse 0.04
TMT B 0.04
OMT Acc Easy -0.03
OMT Misbinding -0.02
ROCF Remember 0.02
OMT Acc -0.02
OIS LTM IdeRT -0.02
OMT LocErr Easy 0.02
OMT Acc Hard -0.02
OMT IdeRT 0.01
OMT TargetDetection -0.01
DSST RT -0.01
OIS STM Acc 0.01
ROCF CopyMoveN 0.00
OIS LTM LocRT 0.00
TMT Connect2 -0.00
0
0.05
0.1
0.15
0.2
0.25
0.3
0.35
Absolute Correlation Strength (|Spearman's rho|)

Supplementary Table 3: Spearman correlations between screen height and OCTAL metrics.

Metric	rho	pValue	Significance
ROCF_CopyScore	0.30	0.0004	***
OIS_STM_IdeRT	-0.22	0.05	*
ROCF_CopyDuration	-0.21	0.08	
ROCF_RecallDuration	-0.17	0.48	
TMT_BdA	0.16	0.61	
TMT_A	-0.14	1.00	
ROCF_RecallScore	0.11	1.00	
DSST_pHit	0.11	1.00	
age	0.11	1.00	
OIS_STM_LocRT	-0.11	1.00	
OIS_LTM_LocErr	-0.10	1.00	
ROCF_RecallMoveN	-0.09	1.00	
OMT_LocRT	-0.09	1.00	
OIS_LTM_SemanticAcc	-0.07	1.00	
OIS_STM_LocErr	-0.07	1.00	
OIS_STM_SemanticAcc	0.06	1.00	
OIS_LTM_Acc	-0.06	1.00	
OMT_LocErr	0.05	1.00	
OMT_LocErr_Hard	0.04	1.00	
DSST_nCorrectResponse	0.04	1.00	
TMT_B	0.04	1.00	
OMT_Acc_Easy	-0.03	1.00	
OMT_Misbinding	-0.02	1.00	
ROCF_Remember	0.02	1.00	
OMT_Acc	-0.02	1.00	
OIS_LTM_IdeRT	-0.02	1.00	
OMT_LocErr_Easy	0.02	1.00	
OMT_Acc_Hard	-0.02	1.00	
OMT_IdeRT	0.01	1.00	
OMT_TargetDetection	-0.01	1.00	
DSST_RT	-0.01	1.00	

OIS_STM_Acc	*0.01*	*1.00*	
ROCF_CopyMoveN	*0.00*	*1.00*	
OIS_LTM_LocRT	*0.00*	*1.00*	
TMT_Connect2	*0.00*	*1.00*	

Supplementary Figure 4: Two-factor structure derived from an exploratory factor analysis of 19 OCTAL metrics obtained remotely from 1,109 healthy participants across the adult lifespan (Study 2).

Item labels show task abbreviation and metric; their order is arbitrary. Bar colour indicates the sign of the factor loading (pink = negative, green = positive). Factor 1 (memory) is dominated by high loadings from ROCF Recall, OMT, OIS, and ALF measures of both accuracy and localisation, whereas Factor 2 (executive function) loads chiefly on DSST and TMT-A indices.

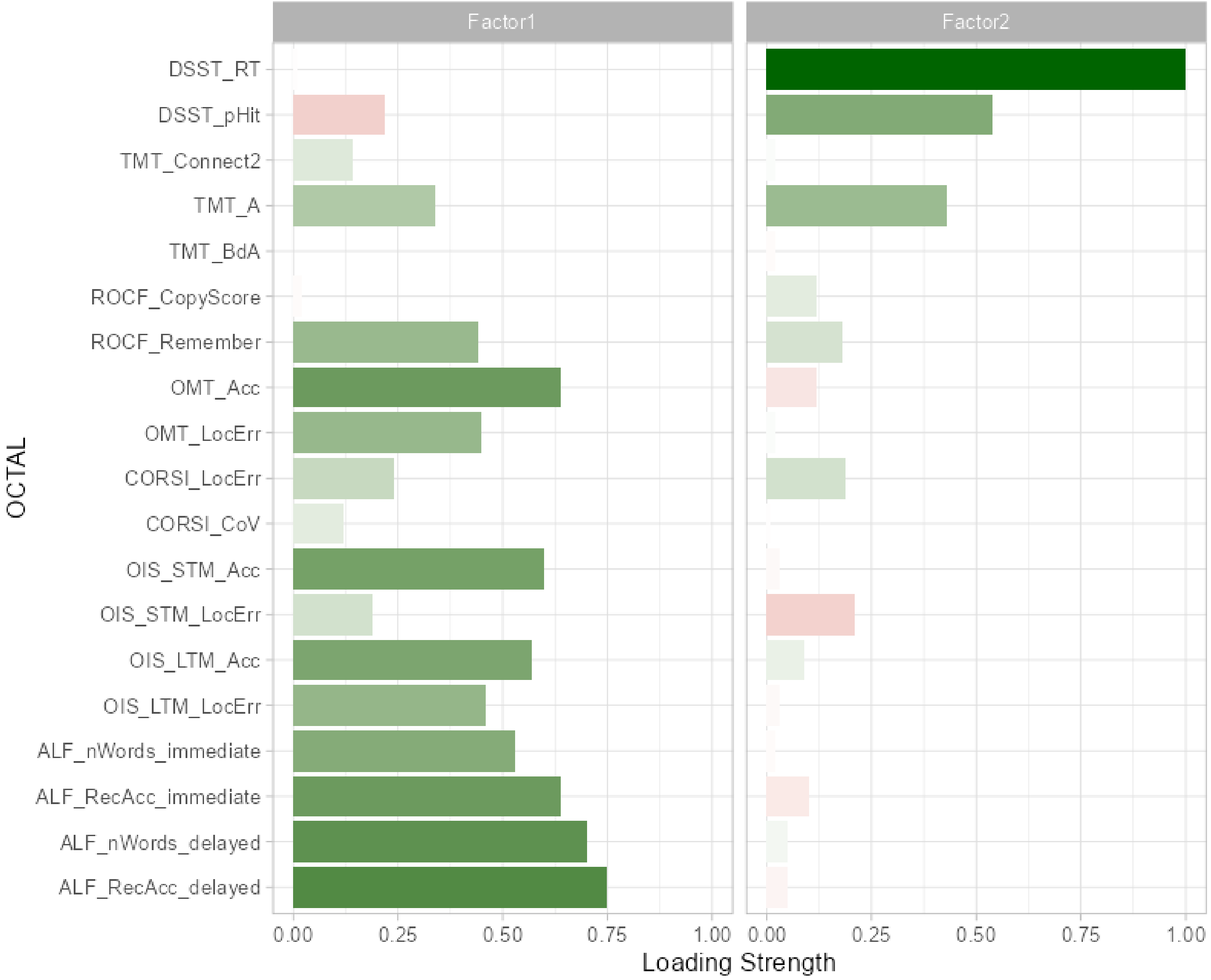

Supplementary Table 4: Spearman correlations between age and OCTAL metrics in Study 2.

Metric	N	rho	p	Significance
TMT_A	478	0.46	p < 0.001	***
TMT_B	478	0.40	p < 0.001	***
DSST_nCorrectResponse	987	-0.39	p < 0.001	***
OMT_IdeRT	420	0.39	p < 0.001	***
TMT_Connect2	478	0.33	p < 0.001	***
OIS_STM_IdeRT	1039	0.31	p < 0.001	***
OMT_LocRT	420	0.28	p < 0.001	***
ROCF_RecallScore	1102	-0.27	p < 0.001	***
OIS_LTM_Acc	1037	-0.26	p < 0.001	***
OIS_LTM_SemanticAcc	1037	-0.25	p < 0.001	***
CORSI_LocErr	384	0.24	p < 0.001	***
ROCF_CopyScore	1101	-0.23	p < 0.001	***
ROCF_Remember	1101	-0.19	p < 0.001	***
ROCF_Remember	1101	-0.19	p < 0.001	***
ALF_RecAcc_immediate	198	0.19	p = 0.009	
OIS_LTM_IdeRT	1039	0.18	p < 0.001	***
OMT_Acc	412	-0.16	p = 0.002	
OMT_Misbinding	412	0.15	p = 0.002	
OMT_Acc_Hard	412	-0.15	p = 0.002	
DSST_pHit	981	-0.15	p < 0.001	***
OIS_STM_LocErr	1039	-0.14	p < 0.001	***
OIS_STM_Acc	1039	-0.14	p < 0.001	***
OMT_TargetDetection	420	-0.11	p = 0.026	
OMT_LocErr_Easy	412	-0.11	p = 0.030	
ALF_nWords_delayed	198	0.10	p = 0.141	
ROCF_RecallDuration	1102	0.10	p < 0.001	*
ALF_nWords_immediate	198	0.09	p = 0.218	
OIS_STM_LocRT	1039	0.09	p = 0.005	
OMT_Acc_Easy	412	-0.08	p = 0.095	
ROCF_CopyDuration	1101	0.08	p = 0.011	
OMT_LocErr_Hard	412	0.07	p = 0.162	

OIS_LTM_LocRT	*1039*	*0.06*	*p = 0.062*	
ALF_RecAcc_delayed	*198*	*-0.05*	*p = 0.521*	
CORSI_CoV	*384*	*-0.04*	*p = 0.444*	
TMT_BdA	*478*	*-0.01*	*p = 0.770*	
OIS_STM_SemanticAcc	*1039*	*-0.01*	*p = 0.823*	
ROCF_RecallMoveN	*1102*	*0.01*	*p = 0.864*	
OMT_LocErr	*412*	*0.00*	*p = 0.928*	
ROCF_CopyMoveN	*1101*	*0.00*	*p = 0.964*	
OIS_LTM_LocErr	*1037*	*0.00*	*p = 0.974*	

Supplementary Figure 5: Age trajectories of OCTAL metrics in a healthy population (Study 2, N = 1,109).

This is additional to Figure 2 in the main manuscript. The top panel displays raw scores for the DSST (A), OIS Short-Term (C) and Long-Term (E) recall, and ROCF memory (G) across ages 45–80 years. Curves are smoothed with a Gaussian kernel (bandwidth = 2.5 years). Marker colour encodes the number of observations contributing to each point, as shown by the colour bar on the right. The bottom panel presents the same metrics normalised to the young-adult reference group (18–39 years). The scale demarcates the normative range (±1 SD, green zone), moderate deviation (1–2 SD, orange zone), and marked impairment (≥2 SD, red zone). Reaction-time variables—such as DSST mean RT in panel A—are sign-reversed so that increasingly negative z-scores indicate greater executive decline. White diamonds along the horizontal white guideline mark age groups that differ significantly from the young-adult mean, whereas black diamonds atop black stems indicate significant deviation from −1 SD (i.e., the boundary between the green and orange zones).

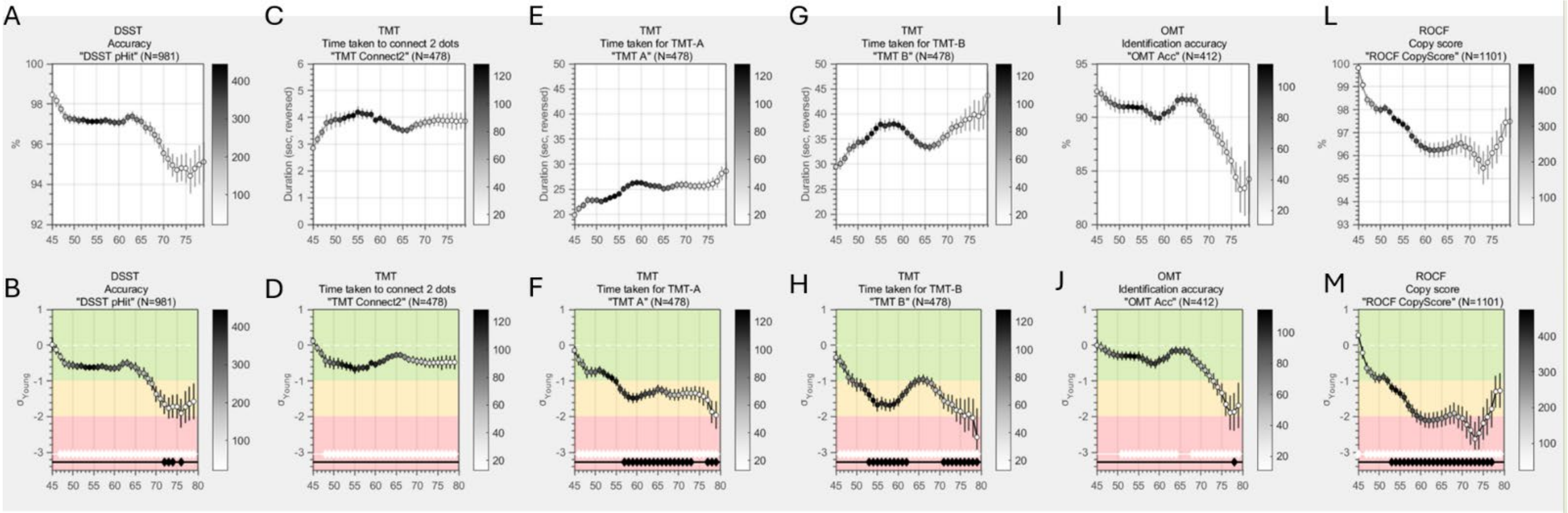

Supplementary Table 5: Slopes of age-effect on OCTAL metrics.

This is supplementary to Figure 3 in the main text.

Metric	Slope (z/year)	R squared
ROCF_CopyScore	-0.07034	0.832969
DSST_RT	-0.0726	0.948665
TMT_Connect2	-0.00924	0.332046
TMT_A	-0.03229	0.666922
TMT_B	-0.02339	0.296664
TMT_BdA	0.010007	0.129192
OMT_Acc	-0.02427	0.493306
OIS_STM_Acc	-0.01469	0.474455
OIS_LTM_Acc	-0.03358	0.683793
OIS_Remember	-0.03115	0.659559
ROCF_Remember	-0.03503	0.811077
ALF_nWords_immediate	-0.00894	0.146788
ALF_nWords_delayed	-0.01987	0.70015
ALF_Remember	-0.01745	0.632817

Supplementary Figure 6. Scatter plots depicting the linear relation between age and each OCTAL metric.

Individual participant scores (N = 1,109) are shown together with least-squares regression lines, providing the numerical slopes that underpin the ranked trajectories in Figure 3. The blue dots showing the kernel-smoothed normalised metrics against age. The red line shows the regression line, with the slope value shown in the title.

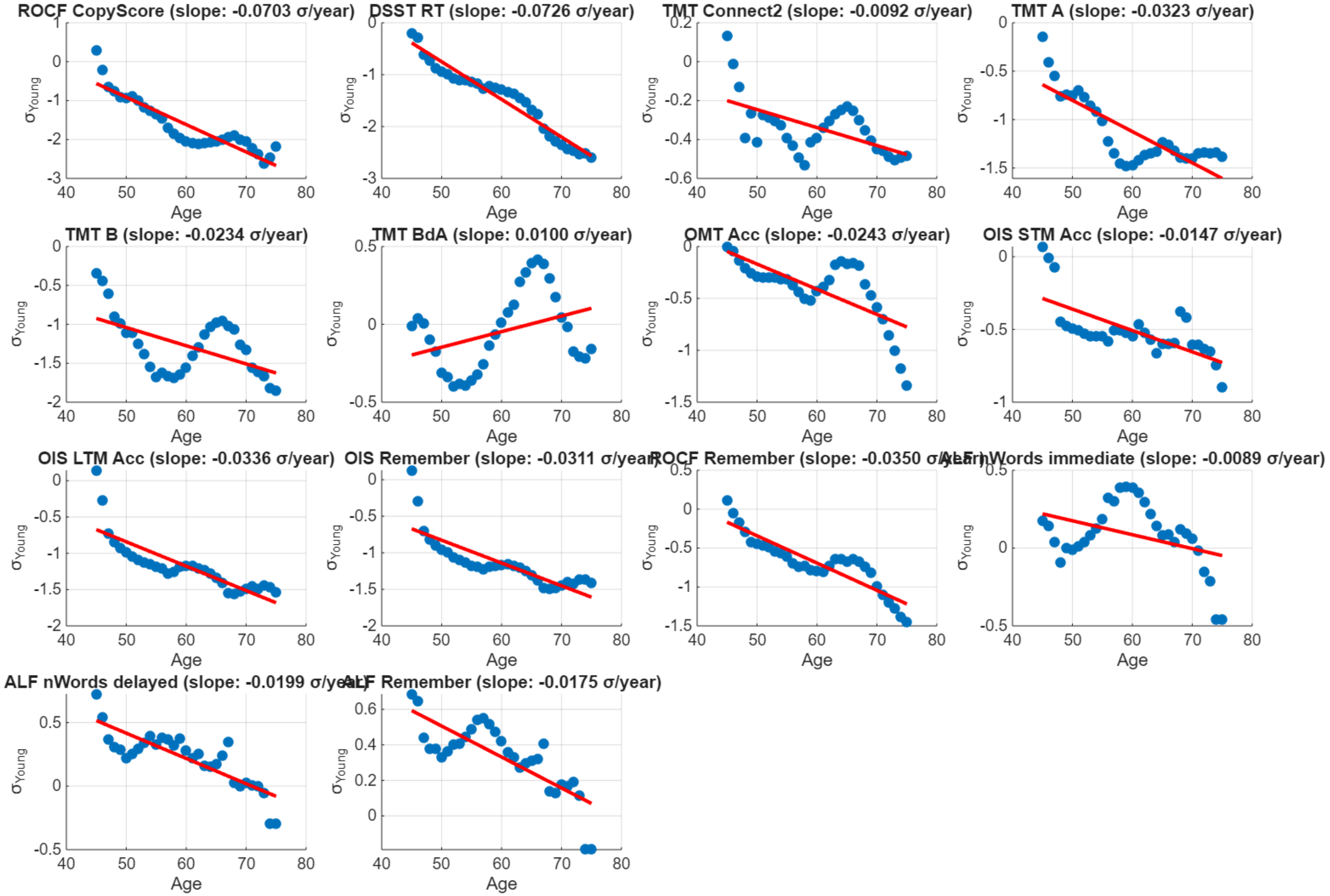

Supplementary Figure 7: Metric-by-metric correlations among OCTAL indices in healthy adults (Study 2), without multiple comparison correction.

The matrix displays Spearman coefficients (ρ) for all pairwise comparisons; only significant correlations ($p < 0.05$) are shown. All metrics are directionally aligned so that lower scores indicate greater cognitive impairment. Square colour, mapped to the accompanying colour bar, encodes the magnitude and sign of ρ, and each cell is labelled with its exact value.

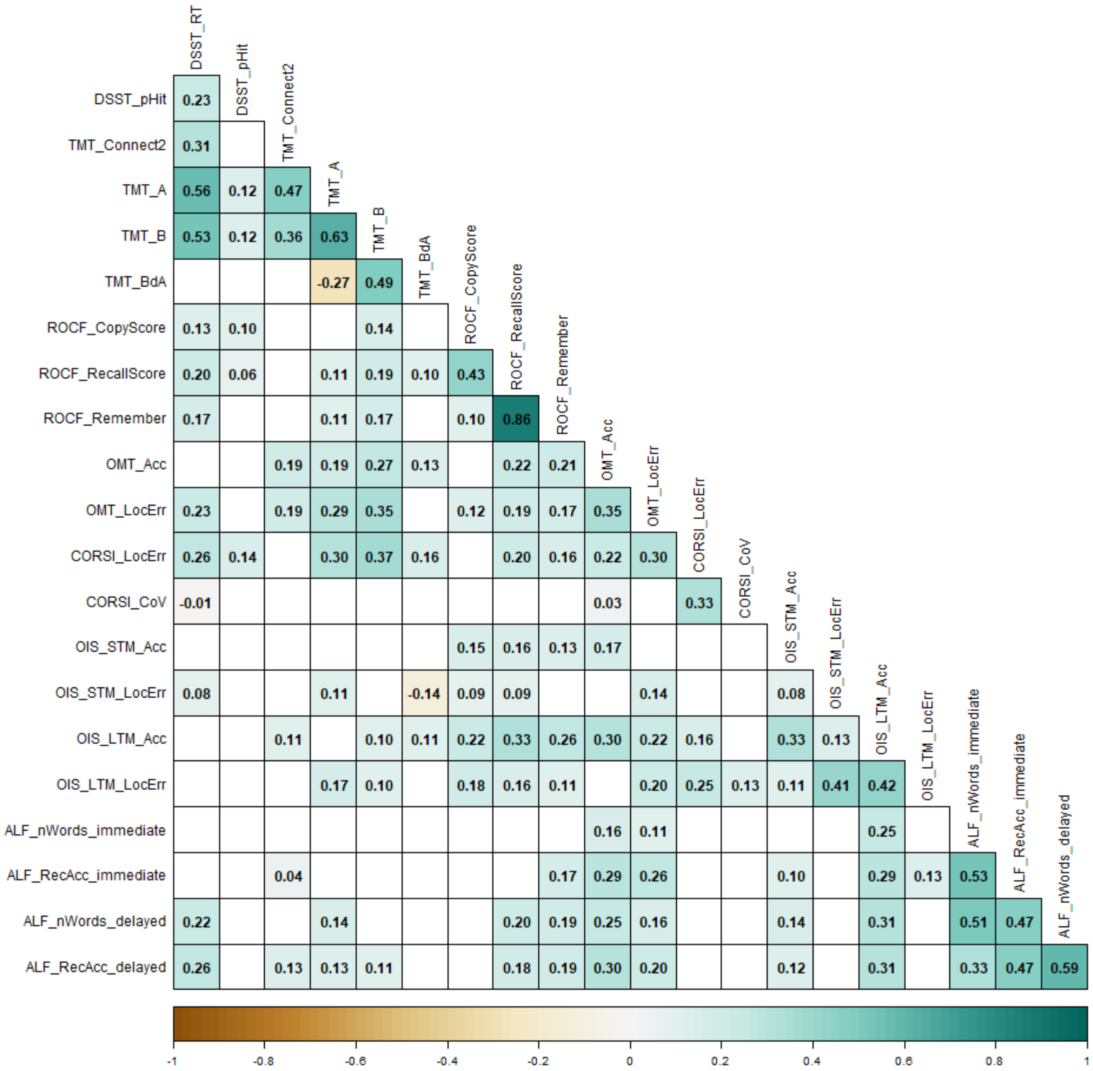
DSST_RT
DSST_pHit
TMT_Connect2
TMT_A
TMT_B
TMT_BdA
ROCF_CopyScore
ROCF_RecallScore
ROCF_Remember
OMT_Acc
OMT_LocErr
CORSI_LocErr
CORSI_CoV
OIS_STM_Acc
OIS_STM_LocErr
OIS_LTM_Acc
OIS_LTM_LocErr
ALF_nWords_immediate
ALF_RecAcc_immediate
ALF_nWords_delayed
ALF_RecAcc_delayed
-1
-0.8
-0.6
-0.4
-0.2
0
0.2
0.4
0.6
0.8
1

Supplementary Table 6 : Correlation table between ACE-III subscores.

All values are Spearman's rho and all significant ($p < 0.001$ after Bonferroni correction).

	ACE-III Attention	ACE-III Memory	ACE-III Visuospatial	ACE-III Fluency	ACE-III Language
ACE-III Attention	1.00	0.65	0.64	0.57	0.44
ACE-III Memory	0.65	1.00	0.61	0.69	0.55
ACE-III Visuospatial	0.64	0.61	1.00	0.56	0.52
ACE-III Fluency	0.57	0.69	0.56	1.00	0.52
ACE-III Language	0.44	0.55	0.52	0.52	1.00

Supplementary Figure 8: ROC for different thresholding for ACE-III

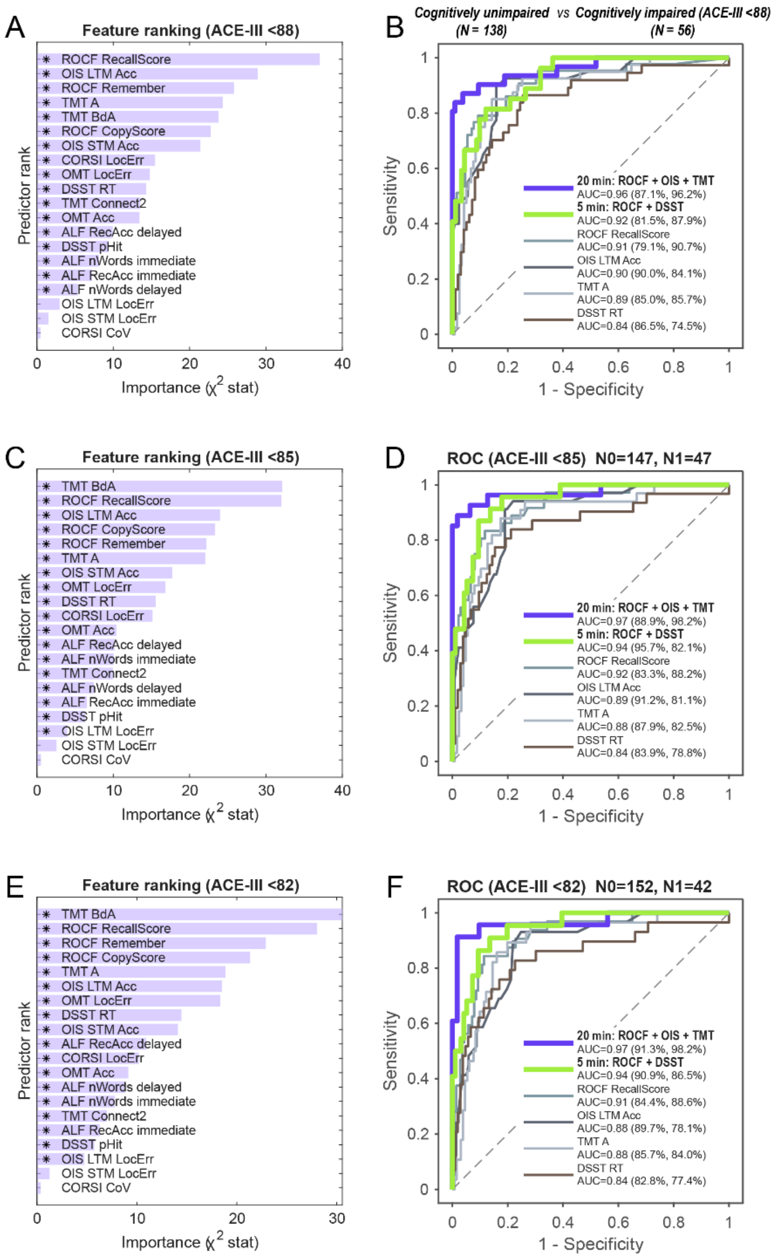

Supplementary Table 7: Consistency across different thresholding for ACE-III and task combinations for OCTAL.

ModelSet	Method	CutoffPair	SpearmanRho
20min	LASSO	88 vs 85	0.952381
20min	LASSO	88 vs 82	0.833333
20min	LASSO	85 vs 82	0.880952
20min	RF	88 vs 85	0.904762
20min	RF	88 vs 82	0.904762
20min	RF	85 vs 82	1
5min	LASSO	88 vs 85	0.942857
5min	LASSO	88 vs 82	0.942857
5min	LASSO	85 vs 82	1
5min	RF	88 vs 85	0.6
5min	RF	88 vs 82	0.6
5min	RF	85 vs 82	1

Supplementary Table 8: Intraclass correlation coefficients (ICCs) for all OCTAL and ACE-III metrics.

Two-sided 95 % confidence intervals are given in square brackets. The accompanying F-statistics test the null hypothesis that the ICC equals 0.75—the threshold for "good" reliability under the interpretative framework of Koo and Li[22] —at an α level of 0.05.

Metric	ICC	F_stat
DSST Accuracy	1.00 [1.00 1.00]	F(111,222) = 162.59, p < 0.001
OMT Acc	0.98 [0.98 0.99]	F(89,178) = 19.01, p < 0.001
TMT BdA	0.98 [0.97 0.99]	F(108,216) = 14.53, p < 0.001
ACE-III Total	0.98 [0.97 0.98]	F(120,240) = 11.86, p < 0.001
TMT B	0.97 [0.96 0.98]	F(108,216) = 10.73, p < 0.001
OMT TargetDetection	0.96 [0.95 0.97]	F(89,178) = 7.70, p < 0.001
ALF nWords delayed	0.96 [0.94 0.98]	F(52,104) = 7.51, p < 0.001
ROCF CopyScore	0.96 [0.95 0.97]	F(148,296) = 7.16, p < 0.001
OMT Guessing	0.95 [0.94 0.97]	F(89,178) = 6.36, p < 0.001
OMT Misbinding	0.95 [0.93 0.97]	F(89,178) = 6.15, p < 0.001
OIS LTM LocErr	0.95 [0.93 0.96]	F(132,264) = 5.89, p < 0.001
ACE-III Memory	0.95 [0.93 0.96]	F(120,240) = 5.42, p < 0.001
ALF nWords immediate	0.92 [0.88 0.95]	F(52,104) = 3.79, p < 0.001
ACE-III Fluency	0.92 [0.89 0.94]	F(120,240) = 3.48, p < 0.001
DSST RT	0.92 [0.89 0.94]	F(110,220) = 3.46, p < 0.001
ACE-III Attention	0.91 [0.89 0.94]	F(120,240) = 3.30, p < 0.001
ROCF RecallScore	0.90 [0.88 0.93]	F(147,294) = 2.92, p < 0.001
OIS STM LocErr	0.90 [0.87 0.92]	F(131,262) = 2.77, p < 0.001
TMT A	0.89 [0.85 0.92]	F(108,216) = 2.55, p < 0.001
ACE-III Visuospatial	0.89 [0.85 0.92]	F(120,240) = 2.48, p < 0.001
CORSI LocErr	0.88 [0.85 0.91]	F(142,284) = 2.41, p < 0.001
ROCF Remember	0.87 [0.84 0.90]	F(146,292) = 2.19, p < 0.001
TMT Connect2	0.87 [0.83 0.91]	F(108,216) = 2.17, p < 0.001
OIS LTM Acc	0.86 [0.82 0.89]	F(133,266) = 1.92, p < 0.001
CORSI CoV	0.85 [0.80 0.88]	F(142,284) = 1.74, p < 0.001
OIS STM Acc	0.82 [0.77 0.86]	F(131,262) = 1.45, p = 0.006
ACE-III Language	0.80 [0.75 0.85]	F(120,240) = 1.32, p = 0.035
OMT LocErr	0.79 [0.72 0.85]	F(89,178) = 1.21, p = 0.139

Supplementary Table 9: The full word corpus used in Verbal Memory Wordlist Recall Task (ALF).

The first row lists all 12 semantic categories. In the main task, one word would be randomly selected from each category.

colour	***animals***	***plants***	***vegetables***	***fruits***	***foods***	***materials***	***tools***	***clothing***	***parts of a building***	***weather***	***occupations***
red	*tiger*	*tulip*	*spinach*	*apple*	*garlic*	*gold*	*spoon*	*shoes*	*wall*	*rain*	*engineer*
orange	*lion*	*rose*	*bean*	*lemon*	*oil*	*silver*	*fork*	*blouse*	*floor*	*snow*	*doctor*
yellow	*horse*	*lily*	*potato*	*banana*	*sugar*	*copper*	*pot*	*pants*	*roof*	*storm*	*lawyer*
green	*dog*	*daisy*	*carrot*	*grape*	*salt*	*wood*	*pan*	*skirt*	*ceiling*	*cloud*	*pilot*
blue	*cat*	*tree*	*lettuce*	*peach*	*tea*	*glass*	*pen*	*shirt*	*window*	*sun*	*teacher*
purple	*cow*	*grass*	*corn*	*orange*	*coffee*	*stone*	*knife*	*socks*	*door*	*fog*	*farmer*
brown	*turtle*	*ivy*	*pepper*	*berry*	*wine*	*plastic*	*cup*	*scarf*	*basement*	*ice*	*driver*
white	*frog*	*bamboo*	*pea*	*melon*	*bread*	*paper*	*bowl*	*hat*	*loft*	*rainbow*	*dentist*
black	*fish*	*mushroom*	*tomato*	*pear*	*milk*	*steel*	*plate*	*tie*	*garden*	*wind*	*nurse*
